# Managing withdrawals and exclusions of study participants in COVID-19-research by NUKLEUS

**DOI:** 10.1186/s12874-025-02526-0

**Published:** 2025-05-29

**Authors:** Heike Valentin, Henriette Rau, Lizon Fiedler-Lacombe, Arne Blumentritt, Ekaterina Heim, Alexander Rudolph, Katrin Leyh, Monika Kraus, Bettina Lorenz-Depiereux, Irina Chaplinskaya, Christian Schäfer, Jens Schaller, Joerg Janne Vehreschild, Melanie Stecher, Margarete Scherer, Martin Witzenrath, Beate Balzuweit, Stefan Schreiber, Thomas Bahmer, Wolfgang Lieb, Steffen Cordes, Wolfgang Hoffmann, Sabine Hanß, Dana Stahl

**Affiliations:** 1 Independent Trusted Third Party of the University Medicine Greifswald, Ellernholzstraße 1-2, Greifswald, 17475 Germany; 2 Helmholtz Zentrum München, Abteilung Molekulare Epidemiologie (AME), Ingolstädter Landstraße 1, Neuherberg, 85764 Germany; 3 Institute for Medical Informatics at the University Medical Centre, Robert-Koch-Straße 40, Göttingen, 37099 Germany; 4 University Medicine Greifswald, Fleischmannstraße 6, Greifswald, 17475 Germany; 5 Deutsches Herzzentrum der Charité Berlin, Institute of Computer-assisted Cardiovascular Medicine, Augustenburger Platz 1, Berlin, 13353 Germany; 6 Klinik I für innere Medizin, Uniklinik Köln, Herderstraße 52-54, Köln, 50931 Germany; 7 Department II of Internal Medicine, Hematology/Oncology, Goethe University Frankfurt, Frankfurt Am Main, Germany; 8 Department of Infectious Diseases, Respiratory Medicine and Critical Care, , Charité - Universitätsmedizin Berlin, Corporate Member of Freie Universität Berlin and Humboldt-Universität zu Berlin, Berlin, Germany; 9 Klinik für Innere Medizin I, Universitätsklinikum Schleswig-Holstein, Campus Kiel, Arnold-Heller-Straße 3, Kiel, 24105 Germany; 10 Innere Medizin I, Universitätsklinikum Schleswig-Holstein, Campus Kiel, Arnold-Heller-Straße 3, 24105 Kiel, Germany; 11 Christian-Albrechts-Universität Zu Kiel, UKSH, Campus Kiel, Haus U25, Niemannsweg 11, Kiel, 24105 Germany; 12 German Center for Cardiovascular Research (DZHK) Main Office (Geschäftsstelle) Berlin, Potsdamer Straße 58, Berlin, 10785 Germany; 13 Institute for Community Medicine, Section Epidemiology of Health Care and Community Health, University Medicine Greifswald, Ellernholzstr. 1-2, Greifswald, 17475 Germany; 14 German Center for Cardiovascular Research (DZHK), Partner Site Greifswald, Greifswald, 17489 Germany

**Keywords:** COVID-19, GDPR, Informed consent, Informational right to self-determination, NAPKON, SARS-CoV-2, Data management, Withdrawal, NUKLEUS, Trusted Third Party

## Abstract

**Background:**

This article describes how withdrawals and exclusions of study participants can be managed in COVID-19-cohort studies by NUKLEUS (German: NUM Klinische Epidemiologie- und Studienplattform), using NAPKON (German: Nationales Pandemie Kohorten Netz). The aim of this manuscript was to describe, how partial withdrawals can be performed so that most of the data and bio-samples can be kept for research purposes.

**Methods:**

The study has taken all signed informed consents (ICs) of study participants into account in order to develop a method how partial withdrawals can be developed and installed. The informed consents, which comprise of mandatory and optional modules were investigated to find out which optional modules can be withdrawn from without withdrawing consent from the whole study.

**Results:**

Withdrawals refer to signed ICs including mandatory and optional modules. Withdrawals can be submitted verbally or in writing, and regarding the IC, as a whole, or only partially. Consequently, implemented withdrawals for NAPKON cohorts comprise partial withdrawals with partial or no data deletion or complete withdrawals with data deletion. Thus, more data is still available for research purpose, which would have been lost without the possibility of partial withdrawals. In NAPKON, a total of 3,97% of the participants have submitted a withdrawal or have been excluded from the study if the inclusion criteria were no longer met.

**Conclusions:**

This manuscript is to the author’s knowledge one of the first article related to withdrawals within COVID-19-studies (NAPKON).

The processes serve as ‘best practice’ examples for planning and establishing withdrawal processes in medical research.

## Background

In medical studies, every study participant has the right of informational self-determination. This is the right of the individual to decide for himself/herself on the disclosure and use of his/her personal data.

This right has been developed from the German Basic Law under Article 2 (1) in conjunction with Article 1 (1) [1], as well as the European General Data Protection Regulation (EU-GDPR) [2] and Article 8 [1] of the European Convention on Human Rights [3].

The study participant signs an Informed Consent [4, 5] (hereinafter also referred to as “IC “), in which he/she agrees to participate in the study under the respective conditions described therein.

Each study IC as well as the associated written patient information sheets need approval by an ethics committee. The goal is to safeguard the participant by making sure that all information about the study is completely understood in detail, also by a layperson, and covered in the IC. An informed consent needs to be given by the study participant before inclusion into the study, so that it can act as secure legal basis. Based on the right of informational self-determination, study participants can withdraw the informed consent to a study in whole or in part at any time, without giving any reasons. Signed ICs must meet certain requirements to be legally valid [2, 4]. If this is not the case, existing inconsistencies in the ICs must be resolved in a timely manner. Study participants, whose IC is not legally valid, must be excluded from the study.

Study participants can withdraw fully or partially from a study at any time. Additionally, participants can be excluded from studies by the study’s responsible clinician, e.g. because they no longer meet the inclusion criteria. These different options can result in various withdrawal and exclusion scenarios and possibly complex constellations.

Currently, there is insufficient information in the literature on withdrawal and exclusion processes in COVID-19-cohort studies. Especially in time-critical research projects in the context of the COVID-19-pandemic, it is important to find answers on how withdrawals and exclusions of study participants can be implemented in a timely manner. Particularly, the legal security of the to-be-implemented processes in partly complex constellations of withdrawals and exclusions must be guaranteed at all times.

In the following, the concept and implementation for the withdrawal and exclusion processes for COVID-19-studies managed by the NUKLEUS infrastructure are presented (NUKLEUS: NUM Klinische Epidemiologie- und Studienplattform). The NUKLEUS infrastructure consists of a clinical research platform, composed of TTP, Clinical Data Management (CDM), Imaging Data Management (DIMA), Biomaterial Management (BIMS), Transfer Organisation (TO), which can be used in a variety of clinical studies. These are accompanied by the Ethics Coordination (EC) and the platforms Epidemiology Core Unit (ECU), Biosample Core Unit (BCU) and Interaction Core Unit (ICU).

The processes within NUKLEUS are developed using NAPKON (National Pandemic Cohort Network, German: Nationales Pandemie Kohorten Netz) [6, 7]. NAPKON is funded by the Federal Ministry of Education and Research (BMBF) in the course of the COVID-19-pandemic and aims to bring together and evaluate diagnostic and treatment strategies. This network includes more than 70 centres, which comprise of university hospitals, non-university hospitals, medical practices and medical care centres. The bundling of competencies and resources shall lead to processes that will ensure the best possible care for those suffering from Severe Acute Respiratory Syndrome-Coronavirus-2 (SARS-CoV-2) and which might also be used in future pandemics (pandemic preparedness). In addition to socio-demographic data, medical data such as symptoms, diagnostic data and therapies are collected for this purpose. Moreover, biosamples are collected, in particular blood and respiratory samples (e.g. swab samples).

The Independent Trusted Third Party of the University Medicine Greifswald (TTP) [8] is responsible for the consent management of the described studies.

The aim of this paper is to get answers to the following questions within the NUKLEUS infrastructure:

1. What types of Informed Consents (ICs) are available?
2. How can study participants withdraw and how are their withdrawals processed?
3. How can withdrawals be implemented in practice and in which processes is data to be completely or partially deleted?
4. May consent modules be withdrawn individually and if so, which modules?
5. How to proceed with biosamples?
6. What percentage of study participants submit a withdrawal?

Ethical and legal considerations are included in answering these questions.

### Methods

#### Study setting and recruitment

There are currently three cohort studies implemented within the framework of NAPKON, where the data management and the biospecimen collection is handled by the NUKLEUS infrastructure.

The three cohort study platforms are as follows:

1. The cross-sector platform (German: Sektorenübergreifende Plattform, SÜP) brings together university hospitals, non-university hospitals, public health authorities, medical practices and medical care centres. (age range of participants: from 18 to 96 years)
2. The high-resolution platform (German: Hochauflösende Plattform, HAP) records severely affected patients at selected university hospitals. (age range of participants: from 20 to 93 years)
3. The population-based platform (POP) includes SARS-CoV-2-infected individuals from three geographically defined areas, approximately 9 months after the acute infection and performs a comprehensive examination with regard to long-Covid symptoms (Ref: PMI:34,642,875). (age range of participants: from 18 to 90 years)

For SÜP and HAP, apart from a few control study participants, SARS-CoV-2 positive study participants are included. The SARS-CoV-2 positive patients are invited by the medical staff to participate in the studies (in SÜP and HAP at the latest one week after a positive polymerase chain reaction test (PCR test) for the detection of the viral infection). For POP, the study participants are not SARS-CoV-2 positive any more by the time they are recruited. Recruitment within POP took place in the way that these people got a letter by post or they were invited by the medical staff of the medical practices or medical care centres.

Recruitment for SÜP and HAP takes place in university and non-university hospitals, medical practices and medical care centres (over 70 study sites).

Each potential study participant is thoroughly informed by a doctor about all aspects of the study, that is the collection, the processing and the scientific use of all data. In addition he/she receives this detailed information as a written patient information sheet [9]. At this stage, the information about possible withdrawals is also given.

The potential study participant has enough time to think about all aspects and to ask questions. Afterwards, he/she fills out and signs either a paper-based IC or an electronic informed consent (eIC) via tablet.

The study centres are encouraged to use as often as possible tablets instead of paper-based ICs. Tablets save time and the susceptibility to errors is greatly reduced. There are no criteria, that tables may not be used. Nevertheless, the acceptance is not always given by the participants (which is not related to the age of the participants). Moreover, sometimes the equipment with tablets is not sufficient in a study centre.

In the case that an adult study participant is incapable of giving consent, there is the option that a legally accepted representative may sign the IC on his/her behalf. Additionally, if a person is unable to give informed consent due to a medically proven comatose state, and there is no such representative available, the patient can be included in the cohorts SÜP and HAP via the so-called “presumed will”. This means that at least one medical doctor completes and signs the IC for the patient, if it can be assumed that the informed consent corresponds to the presumed will of this person. Further assent or the patient’s consent must be obtained immediately after a legally accepted representative becomes available or after the patient regains the capacity to consent.

The concepts and solutions presented in this paper refer only to full withdrawals, partial withdrawals and active study exclusions [2] of adult study participants. The withdrawal and exclusion management of paediatric participants is not considered in this paper.

### IC structure and management

The ICs within the NAPKON studies are modular in design [4, 10], based on current legal and ethical principles and can be amended, if the content needs updates or legal requirements have changed. If this happens, each new version again needs to be approved by an ethics committee. Furthermore, it is sometimes necessary that slightly different IC versions need to be implemented for individual university hospitals or federal states, according to the requirements of the responsible ethics committees.

The structure of an IC consists of introductory information about the study, the informed consent text (privacy statement), optional modules to which the study participant must either clearly agree by ticking “yes” or reject by ticking “no”. Finally, both the study participant and the medical doctor must sign the document. The informed consent text and optional modules currently differ structurally and partially in terms of content for the three cohort platforms SÜP, HAP and POP. Table 1 shows different optional modules for the three cohorts.

Solutions for non-German speaking participants were also offered. Patient information and ICs were translated into English where necessary and implemented as such.

The ICs are stored and managed at the TTP. The software solution gICS (*generic Informed Consent Service*) [4, 10] of the University Medicine Greifswald is used for the consent management. gICS can be integrated into both paper-based and purely digital workflows, and is used to process modular ICs and subsequent withdrawals in the study context.

**Table 1:** Examples of optional IC modules for the three cohorts. Description of the modules is simplified

Cohort study Examples for optional modules	SÜP Version 5.0 ^*^	HAP Version 3.1 ^*^	POP Version 1.9 ^*^
Contact pre-treating medical doctor or general practitioner	Yes	Yes	Yes
Query and use of personal health data (health insurance, pension insurance)	Yes	No	Partial (only health insurance data)
Coded transfer of data to third countries	Yes	Yes	Yes
Re-contact of participants at a later date for additional information and/or biosamples	Yes	Yes	Yes
Re-contact of participants at a later date to communicate additional medical findings	Yes	Yes	Yes
Informed Consent to the collection of biosamples	Separate informed consent available that includes consent to collection of biosamples	collection of biosamples mandatory	collection of biosamples was mandatory
Permission for genetic testing of biosamples	permission given by default	Yes	permission given by default
Provision of data and biosamples for cooperating companies	no	Yes	No

*These are only some chosen versions. The ICs of all three cohorts will be continuously adapted, if necessary, both in terms of content and versioning

### Withdrawals and exclusion from studies

In case the inclusion criteria are not met or the informed consent is not legally valid, a participant needs to be excluded from the study. This decision is made by the study centre.

In addition, based on their own decision, participants have the right to withdraw their participation in the study, in full or in part, at any time and without giving any reason. If a study participant wants to withdraw, the study centre informs the participant about the different possibilities of withdrawals in order to choose the correct one for the specific situation.

Possible reasons for a partial of a complete withdrawal are the following:

- Participant is moving to another city so that the participation in a study would be too difficult.
- Participant does not want to be contacted any more due to time constraints.
- Participant does not want to give any more bio samples.
- Participant decided to completely withdraw as he/she does not feel comfortable any more with participation
- health concerns
- not interested any more as COVID already was exhausting enough

All decisions regarding withdrawals and exclusions from study must be transferred to the TTP, as the TTP is orchestrating these processes. In NUKLEUS medical data, biosamples, images and person identifying data are stored in different places, therefore each withdrawal or study exclusion must be carried out at all of the following infrastructure partners:

1. Clinical Data Management (CDM): The Institute for Medical Informatics at the University Medical Centre in Göttingen administrates and stores medical data using secuTrial [11].
2. DICOM Data Management System (DIMA): The Charité – Universitätsmedizin Berlin maintain the DIMA for the central documentation and quality assurance of DICOM data (Digital Imaging and Communications in Medicine) using TrialComplete [11].
3. Biobanking Information Management System (BIMS): Supports the processing of biosamples and the associated workflows by providing a laboratory information and management system (LIMS/CentraXX)

### Development of the concept and parameters used for the evaluation

The concept and workflows for processing and implementing withdrawals and study exclusions in COVID-19-research within the NAPKON framework were developed by the NUKLEUS infrastructure, mainly by the Clinical Data Management, BIMS and DIMA, the Ethics Coordination and the members of the study coordination of SÜP, HAP and POP. Already existing processes of the German Centre for Cardiovascular Research (DZHK) served as a basis. Fundamental structures and objectives for all processes regarding withdrawals and study exclusions were defined. Based on these results, further discussions and workshops were held over several months with scientists from the fields of biobanking, ethics, human genetics, information management, information research, image data management and clinical studies.

Those workshops operated as informal discussion groups. All partners of the NUKLEUS community who were interested in giving insights as experts in their field as well as feedback to concepts, were openly invited. Discussions took place in small groups and always concerning a defined topic.

The aim of these workshops was to define differentiated withdrawal and exclusion processes that cover the possible and necessary constellations/scenarios.

The practicability and implementation of the developed concept including workflows and solutions for managing withdrawals and exclusions while considering the complex constellations in NAPKON using the NUKLEUS infrastructure, should also be evaluated. For this purpose, a) a classification of withdrawals and study exclusions was developed, b) the number of recruited study participants was set in relation to the number of study participants with study exclusions or withdrawals and c) the number of those data sets that are retained for research purposes by using partial withdrawals was determined.

## Results

The concept and the processes of withdrawal and exclusion management for the three cohorts SÜP, HAP and POP are based on the different ICs in these studies.

Within NAPKON not all ICs had been approved by an ethics committee for each study site. Therefore, it might happen that only a subset of the possible ICs is available for a particular site.

### Types of informed consents and documents

Different informed consent forms for different scenarios are available for the three cohorts. Additionally, the study participant receives a detailed patient information sheet going along with each IC.

In the following table you see the types of consents per platform.

Whether a biosample collection has been taken place or not, was dependent on two requirements:

- whether the centre was able to collect biosamples.
- Some medical practices or medical care centres did not have the possibility to store and manage these samples for a study.
- whether the participant was willing to consent to the biosample collection

Except for the informed consent for “Presumed Will”, which is currently only used in paper form, all other informed consents can be completed and signed as paper-based version or via tablet (completely electronic informed consent collection). According to Good Clinical Practice (GCP) [9], the study participant must receive a copy of the informed consent. Consequently, the study participant receives a printout of the IC document in case of a fully electronic informed consent collection.

In the HAP-cohort, in contrast to SÜP, only two types of ICs are available:

One study IC is the regular study IC including an optional biosample module, which can be signed by the study participant or a representative. A second version of inclusion via “presumed will” is possible, in which case a medical doctor signs the informed consent.

In the HAP cohort additional ICs for control cohorts have been made available since the beginning of 2022.

In the POP cohort, COVID-19-effects on the study participants’ health status and quality of life are investigated. For this cohort one study informed consent as well as one supplementary informed consent for the second examination are available. These ICs can only be signed by study participants, who are able to give informed consent.

### How withdrawals and study exclusions are communicated to the TTP

Withdrawals by study participants are received and processed by the TTP of the University Medicine Greifswald. There are two options for study participants to withdraw an informed consent, which are available for all three cohorts:

1. The study participant withdraws orally or in writing to the study centre, i.e. in presence of the study medical doctor. This information is filled into a withdrawal form and transmitted as encrypted document to the TTP.
2. The study participant withdraws by writing an e-mail to widerruf@napkon.de (installed for SÜP, HAP, POP). This e-mail is forwarded to the TTP and processed throughout the NUKLEUS IT-infrastructure.

In case of a study exclusion, i.e. not meeting inclusion criteria any more (e.g. false-positive PCR test), a separate study exclusion form is filled out by the study staff in the centre and transmitted to the TTP in encrypted form.

Study exclusions can also be identified by the TTP. After approximately 3 months, study participants’ datasets with still invalid ICs are disabled for data entry and, subsequently, also deleted, if the errors are not immediately corrected. In this case, the TTP communicates the study exclusion to the study centre and processes the exclusion according to the defined workflows.

### Implementing withdrawals and study exclusions in practice and resulting processes

In principle, it would be possible to withdraw each consented module individually in each IC. However, this procedure would mean an immense administrative effort, which is not desirable, especially for time-critical research like in COVID-19-studies. In order to ensure practicability, it was agreed in the NUKLEUS infrastructure that the following withdrawal processes should be technically implemented:

For SÜP and HAP:

- Partial withdrawal without data deletion (Blocking for further biosample collection)
- Partial withdrawal without data deletion (Contact blocking incl. blocking of further biosample collection)
- Partial withdrawal with only deletion of person-identifying data
- Complete withdrawal with data deletion and biosample destruction
- Study exclusion with data deletion and biosample destruction

For POP:

- Partial withdrawal with only deletion of person-identifying data
- Complete withdrawal with data deletion and biosample destruction
- Study exclusion with data deletion and biosample destruction

#### Partial withdrawal without data deletion (Blocking for further biosample collection)

This option includes the stop of further biosample collection (biomaterial collection blocking), i.e. study staff is not allowed any more to collect new biosamples. However, existing biosamples can still be used for research purpose, and the person remains as study participant in the cohort.

#### Partial withdrawal without data deletion (Contact blocking incl. blocking of further biosample collection)

In this option the participant withdraws to be contacted again. For Napkon/NUKLEUS this also includes that no further biosamples are allowed to be collected. Nevertheless, existing biosamples can continue to be used for research purpose. Furthermore, the study participant remains in the cohort.

#### Partial withdrawal with deletion of only person-identifying data

In this case, the study participant wants his/her data to be anonymised.

In the literature, no legally clear solution is described as to how and whether biosamples and images can be anonymised. For this reason, NAPKON could only define the deletion of person-identifying data as a “de facto anonymisation” and cannot assume an absolute anonymisation. The to-be-deleted person-identifying data are: last name, first name, gender, date and place of birth. Consequently, the collected data can no longer be assigned to a specific person by legal means in no part of the infrastructure. Scientists using the data contractually assure not to attempt to identify individuals. Existing biosamples, existing medical and image data are still available for research purposes.

If a study participant was included with “presumed will” and he/she dies before a regular informed consent (where he/she can sign by himself/herself or a legal representative on his/her behalf) could be given, this kind of partial withdrawal is applied as well (only applicable for SÜP and for HAP).

#### Complete withdrawal with data deletion and biosample destruction

If the participation in the entire study is withdrawn, all data, i.e. person-identifying data, medical data, and data concerning biosamples and images, are deleted from all systems and existing biosamples are destroyed at the study centre. Thus, this person is no longer a study participant in the cohort.

#### Study exclusion with data deletion and biosample destruction

A study participant must be definitively excluded, if the inclusion criteria are not met (any more). If a participant is finally excluded from a study, all data will be deleted and existing biosamples will be destroyed by the study centre. Consequently, the person is no longer a study participant in the cohort.

In contrast, there are study participants, who have consented to the study, but no longer come to the agreed follow-up visits. These are referred to as “lost to follow-up” study participants and are documented as such. This situation does not constitute a withdrawal.

**Fig. 1:**
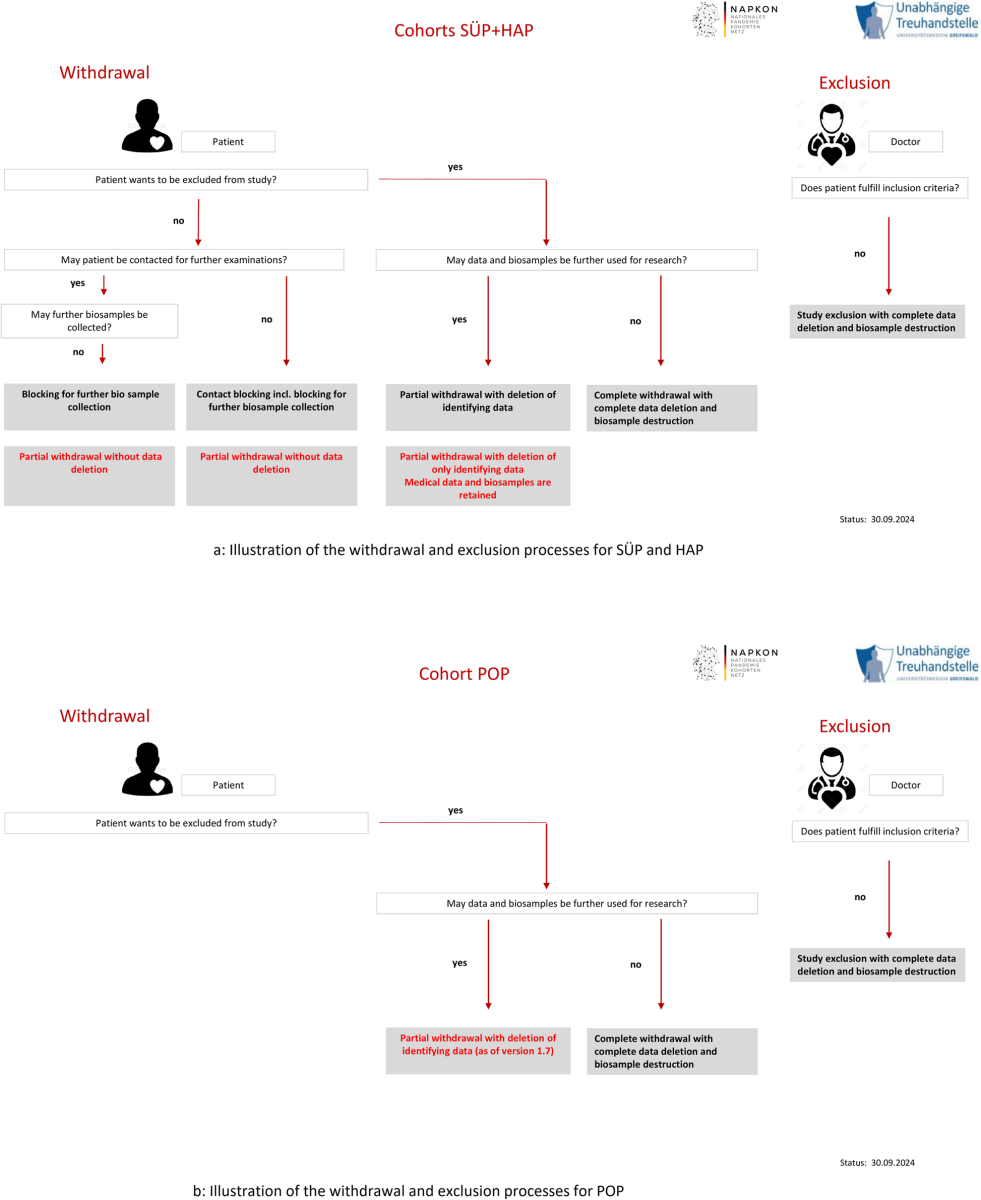
**a** Illustration of the withdrawal and exclusion processes for SÜP and HAP. **b** Illustration of the withdrawal and exclusion processes for POP

In the below Fig. 1a and b you see an overview of the processes for withdrawals and exclusions for the cohorts SÜP/HAP and POP. These include participants capable of giving consent.

### Withdrawal of individual modules

The only optional module within an IC, that can be withdrawn individually is the module for re-contact (“partial withdrawal without data deletion”). In addition, the participant can decide, that no further biosamples may be taken (also a “partial withdrawal without data deletion”). No other individual module can be withdrawn.

If other modules shall be withdrawn, the complete IC must be withdrawn (with deletion of all data and destruction of biosamples).

### Involvement of infrastructure partners of NUKLEUS in data deletion processes

The infrastructure of NUKLEUS includes not only the TTP in Greifswald, but also other partners, who are involved in the described deletion processes. Data deletion for example has to be coordinated between the TTP, Clinical Data Management, BIMS and DIMA. The following Tables 2 and 3 shows the processes in which the infrastructure partners are involved, when deletions need to be done.

**Table 2:** Types of consents per platform SÜP, HAP, POP

Type of consent	Consenting person	Cohort study		
		SÜP	HAP	POP
Basic Consent	for Patient	x		
	for Representatives	x		
	Subsequentely	x		
Basic Consent + Collection of biosamples	for Patient	x		
	for Representatives	x		
	Subsequentely	x		
Consent Presumed Will	Medical Staff	x	x	
Basic Consent + Collection of biosamples + liquor	for Patient	x		
Basic Consent + Collection of biosamples for control cohorts	for Patient + Representative + Subsequentely (all in one IC)		x	
Basic Consent + Collection of biosamples	for Patient + Representative + Subsequentely (all in one IC)		x	
Basic Consent + Collection of biosamples for each study visit	for Patient			x

**Table 3:** Complete deletion or partial deletion at CDM, BIMS, DIMA per withdrawal type

Withdrawal type	Deletion at CDM (clinical data)	Deletion at BIMS (biosample data)	Deletion at DIMA (image data)
partial withdrawal without data deletion (no further biosample collection)	No clinical data deletion	no biosample deletion	no image data deletion
partial withdrawal without data deletion (declining re-contact and no further biosample collection)	No clinical data deletion	no biosample deletion	no image data deletion
partial withdrawal with deletion of person-identifying data. Special case: this process is also applied in case of informed consent for “presumed will”, where the study participant deceases.	No clinical data deletion	no biosample deletion	no image data deletion
Complete withdrawal with data deletion	Clinical data is completely deleted	destruction of biosamples	Image data is deleted
Final exclusion from studies	Clinical data is completely deleted	destruction of biosamples	Image data is deleted

The above table shows that we can offer several options for the participant to withdraw the consent.

Our goal was to create several options, where no data is deleted. This is now the case for three of them.

The choice of these options instead of a complete withdrawal is very desirable as we can here keep all data for later research activities.

### Proportions of study participants, who submit a withdrawal

Table 4 indicates how many study participants per cohort were recruited by 30 September, 2024 and how many partial withdrawals, complete withdrawals and study exclusions were received by the TTP, which have a withdrawal date before September 30th, 2024. (year-to-date).

**Table 4:** Number of withdrawals and study exclusions per total number of study participants (per September 30th, 2024), excluding participants younger than 18 years

	SÜP	HAP	POP	Total of all cohorts
NUMBER OF STUDY PARTICIPANTS INCL. all withdrawals	2619	744	3633	6996
- partial withdrawal without data deletion (no further biosample collection)	6	2	not available for this cohort	8
- partial withdrawal without data deletion (declining re-contact and no further biosample collection)	104	67	not available for this cohort	171
- partial withdrawal with deletion of only person-identifying data	3	0	0	3
- complete withdrawal with complete data deletion	44	7	1	52
- study exclusion with complete data deletion	15	28	1	44
TOTAL WITHDRAWALS and EXCLUSIONS	172	104	2	278
TOTAL WITHDRAWALS and EXCLUSIONS per number of study participants	6,57%	13,98%	0,06%	3,97%

In total, 182 partial withdrawals without data deletion were processed, which is 65,4% of the total number of withdrawals and exclusions (278). When deducting the 44 study exclusions, which are carried out at a time, where no data had been collected yet, we have 182 partial withdrawals out of 234 total withdrawals, which is a ratio of 77,7%.

## Discussion

All withdrawals and exclusions received by the TTP can be classified into the above categories.

With the help of the cascading partial withdrawals, it is possible to give the study participants the opportunity to withdraw without having their medical data/image data deleted and their biosamples destroyed.

This leads to the fact, that the vast majority of data and biosamples can be made available for scientific research.

Especially for the biosamples it is ethically desirable to retain this valuable resource, so that this invasive intervention has not been "in vain".

Furthermore, the medical data and biosamples of study participants who were included with presumed will and deceased are preserved for research. This is assumed to be justifiable, since the medical doctor had exhausted all possibilities when including study participants with “presumed will”, that is, to discuss with relatives, supervisors, the family medical doctor and also to check whether a patient decree was available or whether there was informed consent for other studies.

The majority of withdrawals are recorded and forwarded to the TTP by the study centres, which had been trained in the different withdrawal processes. We therefore assume that we present a realistic number of withdrawals in the single categories.

The percentage of submitted withdrawals in the HAP-cohort is higher than in the SÜP-cohort. Since patients in the HAP-cohort receive intensive medical treatment and are therefore also subject to more elaborate examination programs and closer follow-ups, it can be assumed that they are more inclined to submit a withdrawal. Therefore, especially for the HAP cohort the partial withdrawals are very valuable.

In the POP cohort there have not many withdrawals been processed. This has to do with the fact, that here only patients were recruited, whose Covid-19 infection was at least 6 months ago. They were more willing to participate for a longer time as they were not ill any more.

## Conclusions

This paper is one of the first scientific article on the topic of "withdrawals” in COVID-19-studies (here related to NAPKON^6^) in the literature.

At the TTP of the University Medicine Greifswald all withdrawal processes within the NUKLEUS infrastructure for the cohorts SÜP, HAP and POP are implemented according to this concept. The concept can be understood as "best practice" and serves as a basis for rethinking and redesigning withdrawal processes in medical cohort studies, so that the highest priority can be given to the will of the study participant, while simultaneously retain as much data and biosamples for research purpose as possible. Therefore, our results support re-using the presented concepts for other studies.

The design of withdrawal processes must always be integrated into current legal and ethical contexts. For this reason, the above-mentioned processes are subject to a constant review.

Due to the original diversity in the study documents of SÜP, HAP and POP, it has become apparent that different phrasings and processes have developed per study over time. The further harmonisation of the documents for the study participants related to the presented withdrawal processes is in progress and should also be applied in future new studies within the NUKLEUS/NAPKON structure.

## Data Availability

gICS [12] is available as download via the official website of the Trusted Third Party of the University Medicine Greifswald [12] and from the corresponding author on reasonable request. The data (Informed Consents) underlying this article cannot be shared publicly due to the privacy of individuals (Informed Consents include sensitive data) participated in the study.

## Abbreviations

BIMS: Biobanking Information Management System
CDM: Clinical Data Management
DIMA: DICOM Data Management System
DZHK: German Center for Cardiovascular Research (Deutsches Zentrum für Herz-Kreislauf-Forschung)
EC: Ethics Coordination
eIC: Electronic informed consent
EU-GDPR: European General Data Protection Regulation
GCP: Good Clinical Practice
HAP: Hochauflösende Plattform
IC: Informed Consent
NAPKON: National Pandemic Cohort Network, German: Nationales Pandemie Kohorten Netz
NUKLEUS: NUM Klinische Epidemiologie- und Studienplattform
POP: Populationsbasierte Plattform
SÜP: Sektorenübergreifende Plattform
TTP: Trusted Third Party Declarations
